# Extra-skeletal Ewing Sarcoma of the chest wall in a child

**DOI:** 10.4102/sajr.v23i1.1733

**Published:** 2019-06-27

**Authors:** Denny Mathew, Daniel N. Prince, Nasreen Mahomed

**Affiliations:** 1 Diagnostic Radiology, University of the Witwatersrand, Johannesburg, South Africa; 2 Department of Radiology, Rahima Moosa Mother and Child Hospital, University of the Witwatersrand, Johannesburg, South Africa; 3 South African Society of Paediatric Imaging (SASPI), Cresta, South Africa

**Keywords:** Extra-skeletal, Ewing Sarcoma, tumour, Malignant Paediatric Chest Wall Lesion, computed tomography

## Abstract

Chest wall or pleural-based tumours represent a heterogeneous group of lesions that are infrequent in children and infants; however, a large proportion of these lesions are malignant in nature. Categorising them on the basis of primary versus secondary, site of origin (osseous and cartilage, or soft tissue) and tissue composition may assist in narrowing the differential diagnosis. We present a case of a 7-year-old boy with a progressive history of dyspnoea. The initial chest radiograph (CXR) demonstrated complete opacification of the left hemithorax with no air bronchograms. This was associated with the cut-off of the left main bronchus and mediastinal shift to the right. The post-contrast computed tomography (CT) of the chest showed multiple left-sided enhancing pleural-based masses with collapse of the left lung. These lesions were locally invasive as demonstrated by the intra and extra-thoracic extension. There were no associated erosions of the adjacent ribs or intra-tumoural calcifications. Based on the imaging findings, the diagnosis of extra-skeletal Ewing sarcoma (ES-EWS) of the chest wall was made with a differential diagnosis of rhabdomyosarcoma. A core biopsy was performed of the pleural-based mass, and histology with immunohistochemistry confirmed the diagnosis of a malignant small round blue cell tumour; subtype Ewing sarcoma family tumour (ESFT). The child was subsequently commenced on chemotherapy. The diagnosis of ES-EWS should be considered when a child or adolescent presents with an ill-defined, eccentric, chest wall mass in the absence of a lesion with a primary osseous origin. Imaging plays a key role in tumour staging, therapeutic planning and follow-up of patients.

## Introduction

Ewing sarcoma family tumours (ESFT) are a group of malignant small round blue cell tumours with varying degrees of neuroectodermal differentiation.^1^ They include Ewing sarcoma of the bone (EWS), extra-skeletal Ewing sarcoma (ES-EWS), peripheral primitive neuroectodermal tumour (pPNET) and Askin tumour.^2,3,4^ Despite the contradictory terminology in the literature, this group of tumours is believed to represent the same entity that is histogenetically related, with minor differences in their differentiation.^1,2^ They are associated with a non-random, reciprocal translocation between chromosomes 11 and 22, t(11;22) (q24;q12), with the resultant formation of EWS E26 transformation specific-fusion gene.^2,3,5,6^ This report aims to highlight an entity that is uncommonly reported in the literature, with an approach to other differential considerations for a locally invasive pleural-based/chest wall lesion in a child.

## Case report

**FIGURE 1:**
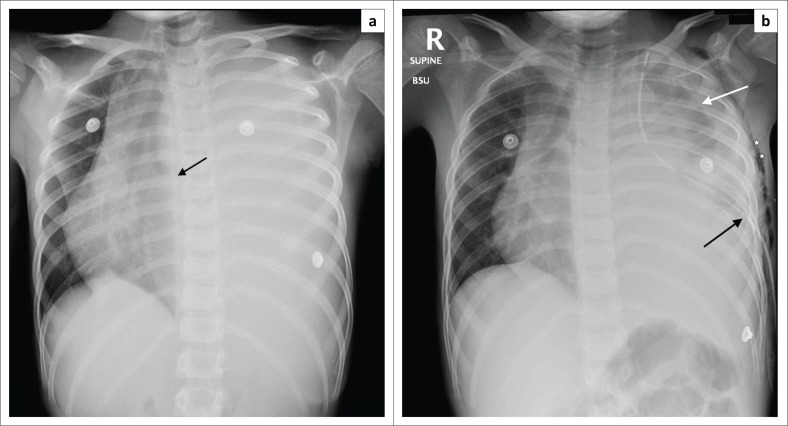
Frontal chest radiograph (CXR): (a) Initial CXR on presentation demonstrates complete opacification of the left hemithorax with resultant mediastinal shift to the right and cut-off of the left main bronchus (black arrow); (b) CXR post left chest wall/pleural biopsy. Left pneumothorax (white arrow) with the left intercostal drain in-situ (black arrow). Associated surgical emphysema of the left chest wall (white stars).

**FIGURE 2:**
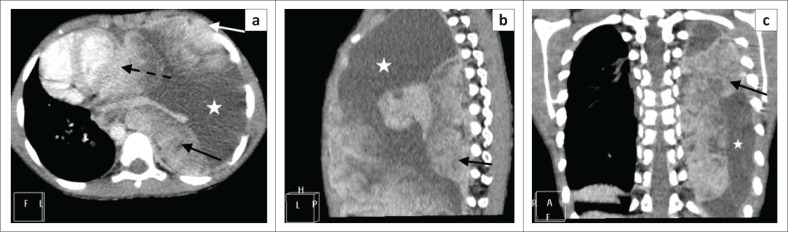
(a–c) Axial, sagittal and coronal contrast-enhanced computed tomography images demonstrate left pleural-based masses which enhance heterogeneously (solid black arrows) with patchy hypodense regions suggestive of necrosis. There is an associated large complex left pleural effusion (white star), atelectasis of the left lung and mediastinal shift to the right. There were no associated intra-tumoural calcifications. Figure 2A – Note the extra-thoracic extension of the mass anteriorly via the intercostal spaces (white arrow) and another pleural-based mass that is inseparable from the pleural pericardial reflection (broken black arrow).

**FIGURE 3:**
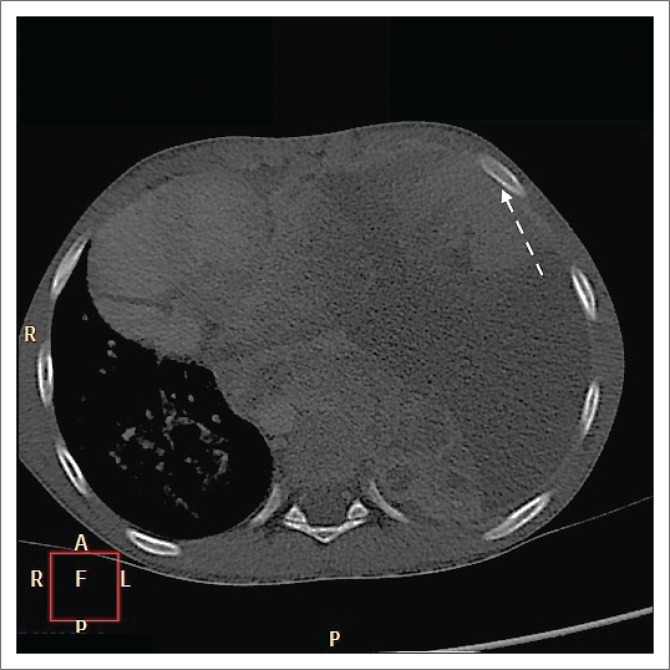
Axial computed tomography image of the chest on bone window demonstrates the absence of associated bony erosions (white arrow).

A 7-year-old boy presented with a progressive history of dyspnoea. A chest radiograph (CXR) was performed which demonstrated complete opacification of the left hemithorax with no air bronchograms. This was associated with a cut-off of the left main bronchus and mediastinal shift to the right (Figure 1a and b). An ultrasound (US) showed multiple pleural-based masses with heterogeneous echo texture and an associated complex left pleural effusion. A computed tomography (CT) scan of the chest with intravenous contrast agent demonstrated multiple ill-defined enhancing left pleural-based masses, with patchy hypodense areas (Figure 2a–c). There were no associated intra-tumoural calcifications. These pleural-based masses were locally invasive, as evident by the poor planes of separation between the pericardium (Figure 2a), descending thoracic aorta (which was shifted to the right of the vertebral column), and diaphragm. The findings noted on CXR of the left lung collapse with a cut-off of the left main bronchus and mediastinal shift to the right were also evident. The extra-thoracic extension was also noted anteriorly via the intercostal spaces (Figure 2a). There were no associated erosions of the adjacent ribs (Figure 3) nor extension of the mass into the vertebral canal. A large complex left pleural effusion (25 Hounsfield units [HU]) was also present, as previously noted on the US scan. The right lung and chest wall were normal, with no pulmonary nodules or masses to suggest metastases.

Based on the imaging findings, the differential diagnosis was categorised into three main groups:

- Malignant soft tissue tumour (rhabdomyosarcoma, malignant peripheral nerve sheath tumour [MPNST], type II/III pleuropulmonary blastoma)
- ESFT
- Metastases (rhabdomyosarcoma, neuroblastoma, lymphoma, leukaemia)

Another differential consideration includes inflammatory myofibroblastic tumour (IMT) of the lung or pleura.

Biopsy of the anterior pleural-based mass was performed for histopathological confirmation. On microscopy, the specimen was composed of a proliferation of small round blue cells arranged in solid sheets. The background stroma was myxoid to oedematous in areas. Large areas of haemorrhage were also noted. The individual neoplastic cells were intermediate in size. The cells demonstrated oval nuclei with irregular contours and finely dispersed chromatin. Pinpoint nuclei were also noted in other areas. The cells had moderate amounts of pale eosinophilic cytoplasm. Brisk mitotic activity was noted with atypical forms seen. No rosettes were identified and a fibrillary background was not identified. No rhabdoid cells were seen. Periodic Acid-Schiff (PAS) and Periodic Acid-Schiff with diastase (PAS-D) stains highlighted focal glycogen within the neoplastic cells. These features were consistent with a malignant small round blue cell tumour of the chest wall/pleural biopsy.

The immunohistochemistry of the biopsy specimens was then performed to further subtype this neoplasm, which were consistent with ESFT.

## Ethical consideration

No patient identifiable information has been presented.

## Discussion

J. Ewing first described EWS in 1921, and it is now believed to be the second-most common malignant bone tumour in children and adolescents after osteosarcoma.^6^ The origin of EWS from structures other than bone is considered rare and is termed ES-EWS.^1^ In 1969, Tefft et al. initially described an extra-osseous, paravertebral soft tissue, small round cell tumour with morphological features similar to EWS.^7^ Angervall and Enzinger in 1975 later established the entity ES-EWS in their review series of 39 cases of extra-osseous examples of EWS.^7^

The most common sites of occurrence of ES-EWS are the paravertebral region, lower extremities, chest wall, retroperitoneum, pelvis and hip, upper extremities, and head and neck region.^2^ The lesion within the paravertebral region may either be intradural extramedullary or evident as an extradural mass.^2^ Although ES-EWS is primarily a soft tissue tumour, it may invade adjacent osseous structures.^6^ Some authors use the term Askin tumour for the specific localisation of ES-EWS within the chest wall.^4,5^

Extra-skeletal Ewing sarcoma typically manifests in children and young adults, with 85% of the reported cases being between 20 months and 30 years of age.^2^ The radiological evaluation of these patients is multimodal; however, the imaging features may be non-specific.^2^ The imaging findings described below are specific to ES-EWS of the chest wall in conjunction with our case presented. It may present as a solitary or multiple extra-pulmonary masses, demonstrating a pattern of eccentric growth along the chest wall.^3,5^ Other reported findings include pleural effusions, lymphadenopathy, lung and bone metastases.^3^ The expansion of this chest wall lesion may result in the secondary collapse of the lung, or it may directly invade the lung parenchyma.^5^ A paravertebral origin of this lesion may result in extension through the vertebral foramina and can cause secondary bony changes of the adjacent vertebral body.^2,5^

Ultrasound (US) offers the advantages of absence of ionising radiation, easy accessibility and a dynamic study that can be performed at the patient’s bedside, which can also guide needle-biopsy for the histological diagnosis. The US shows that these chest wall masses are often hypoechoic but can have anechoic regions if they are necrotic or haemorrhagic.^2^ Doppler interrogation of the masses reveals associated vascular flow.^2^ Commonly, an associated pleural effusion may be demonstrated.^2^

The CT findings depict an ill-defined, large, unilateral, soft-tissue chest wall mass, with heterogeneous enhancement post contrast.^2^ Low-attenuation regions within the lesion may also be apparent and are representative of haemorrhagic or necrotic areas.^2^ Associated calcifications are rare, as evident in our case, and have been reported in 10% of the cases.^2^ Associated rib destruction has been reported in 25% – 63% of the cases.^2^

The magnetic resonance imaging (MRI) findings, although non-specific, offer the added advantage in better delineating the margins of the mass and assessing its relationship with the surrounding structures and tumour staging.^2,8^ The MR depicts an ill-defined, eccentric soft-tissue mass with intermediate signal on T1 weighted imaging (T1WI) and T2 weighted imaging (T2WI) demonstrating a high signal intensity lesion.^2,5^ Areas of haemorrhage or necrosis may be evident as regions of high signal intensity on T1WI and T2WI compared to the skeletal muscle.^2^ Gadolinium administration results in intense enhancement of the solid components of the tumours and is representative of its hyper vascular nature.^2,5^ These tumours are often locally invasive with direct invasion of the musculature of the chest wall, mediastinum and lung, with subsequent lung collapse, as evident in our case.^2^

Fluorodeoxyglucose Positron Emission Tomography (FDG PET) demonstrates increased radionuclide uptake and may be used in the detection of the primary lesion, residual or recurrent tumour or metastatic lesions.^2^ The use of other nuclear studies such as Indium 111 (^111^In) penetetreotide and Technetium 99m (^99m^Tc) setamibi (MIBI) scans also offer promise in the evaluation of treatment response after surgical resection and chemotherapy.^2^

Histologically, ESFT are small round blue cell tumours – a malignant group of neoplasms with a wide range of differentials.^1^ This should be differentiated from other examples of small round blue cell tumours which may also occur in the chest wall such as neuroblastoma, embryonal rhadomysosarcoma and lymphoma.^6^ The diagnosis is based on the combination of both the clinical and imaging features, as well as the immunohistochemical staining and cytogenetic analysis.^6^ Definitive diagnosis is made with Fluorescence in-situ hybridisation (FISH) to detect the rearrangement of the 22q12 (EWSR1 gene).^1^

Malignant paediatric chest wall tumours represent a diverse group of lesions; however, categorising them on the basis of primary versus secondary, site of origin (osseous and cartilage, or soft tissue) and tissue composition may assist in formulating the differential diagnosis.^8^ In the presence of a primary oncological diagnosis, metastatic lesions such as neuroblastoma, rhabdomyosarcoma and lymphoma/leukaemia need to be excluded.^9^ Metastatic lesions to the chest wall typically present on imaging as lytic bone lesions with associated cortical disruption and periostitis.^9^ Neuroblastoma may present as a posterior mediastinal mass with direct invasion of the chest wall and may extend into the adjacent neural formamina.^9^ Lymphoma is often associated in those with a compromised immune system [e.g. Human Immuno Deficiency Virus/Acquired Immunodeficiency Syndrome (HIV/AIDS), or immunosuppressive therapy].^5^ The patient in this case report was not immune-compromised; so, the diagnosis of lymphoma was considered unlikely. The absence of constitutional symptoms in conjunction with the associated negative biochemical and bone marrow findings excludes leukaemia. The absence of a lesion with primary osseous involvement excludes EWS of the bone and osteosarcoma; in addition, osteosarcoma is typically associated with centrally dense calcifications.^2,9^ Rhabdomyosarcoma of the chest wall cannot reliably be differentiated on imaging from ES-EWS, as both demonstrate non-specific findings of an aggressive chest wall lesion.^2^ Bone involvement typically occurs in the late stage of the disease but is reported in ~20% of patients.^9^ Embryonal rhabdomyosarcoma is a subtype which is unique to the paediatric population.^10^ Malignant peripheral nerve sheath tumours arise within the nerve sheaths and may either occur sporadically or within plexiform neurofibromas in patients known with neurofibromatosis type 1 (NF1).^9^ The presence of a mass lesion that courses along the expected location of a peripheral nerve favours the diagnosis of a peripheral nerve sheath tumour and may be associated with underlying erosions of the adjacent bone.^9^ Pleuropulmonary blastomas typically affect infants and children (overall median age of 4 years).^11^ Type II and III pleuropulmonary blastomas have been reported in older patients, and Type I (purely cystic) pleuropulmonary blastomas exclusively occur in those younger than 3 years of age.^12^ Type III (purely solid) pleuropulmonary blastomas are typically well-circumscribed with heterogeneous enhancement, and although primarily intra-thoracic (arising from the lung or pleura), it may involve the chest wall.^9^ Type II (mixed cystic and solid) pleuropulmonary blastomas are less aggressive and rarely involve the chest wall.^9^

Inflammatory myofibroblastic tumour of the lung usually appears as a solitary, well-marginated, peripheral mass on CXR with heterogeneous enhancement on CT.^13^ Calcifications occur more commonly in children compared to adults.^14^ Pleural IMT is exceedingly rare, with only a few published case reports, and may appear locally invasive with the infiltration of the adjacent chest wall.^13^

As with all oncology cases, the mainstay of treatment is multidisciplinary. Biopsy is necessary to confirm the diagnosis after which neoadjuvent chemotherapy is initially used with the aim of decreasing the size of the primary lesion and eliminating micrometastases.^2^ The primary method for local control is surgical; however, this is dependent on various factors based on the tumour staging.^2^ This would include factors such as patients’ age, tumour size, location and extent of involvement of adjacent vital structures; presence of encasement of adjacent neurovascular bundles and whether the primary lesion can be completely excised with acceptable margins.^2^

The thoraco-pulmonary location of ES-EWS is associated with a poorer prognosis in comparison to ES-EWS at other sites with the 2- and 6-year survival rates reported as 38% and 6%.^2^ Recurrence has also been reported in more than 50% of the cases and may be evident as local recurrence, mediastinal lymphadenopathy or metastatic lesions.^2^

## Conclusion

Extra-skeletal Ewing sarcoma is regarded a rare entity that has not been commonly reported but is an important consideration, given the poor prognosis. This diagnosis should be considered when a child or adolescent presents with an ill-defined, eccentric, chest wall mass, in the absence of a lesion with a primary osseous origin. The differential diagnosis should also include rhabdomyosarcoma of the chest wall, as this cannot reliably be differentiated from ES-EWS on imaging. Imaging plays a key role in tumour staging, therapeutic planning and follow-up of these patients.
